# Water intake during drinking does not alleviate hangover symptoms

**DOI:** 10.3389/fphar.2026.1852528

**Published:** 2026-06-15

**Authors:** Hiromitsu Imai, Satoshi Kushio, Keiko Matsuura, Akihiro Nakamura, Kazumasa Mori, Masafumi Kadowaki, Ichiro Oikawa, Naoto Uemura

**Affiliations:** 1 Department of Medical Ethics, Oita University Faculty of Medicine, Oita, Japan; 2 Clinical Pharmacology Center, Oita University Hospital, Oita, Japan; 3 Research Laboratory, Sanwa Shurui Co., Ltd, Usa, Japan; 4 Department of Biomedicine, Oita University Faculty of Medicine, Oita, Japan; 5 Department of Clinical Pharmacology and Therapeutics, Oita University Faculty of Medicine, Oita, Japan

**Keywords:** chaser, ethanol metabolism, hangover, hangover effect, water

## Abstract

**Background:**

Hangovers can impair the ability to drive a car or work in industrial settings, in addition to causing unpleasant symptoms such as headaches and fatigue. As for reducing hangover symptoms, the effect of consuming water between drinks, often referred to as a “chaser,” has been empirically recognized; however, this effect has not been verified to date. The purpose of this study was to clarify whether intermittent drinking of water during alcohol consumption affects the ethanol and acetaldehyde kinetics in the body and whether it alleviates hangover symptoms with the psychomotor function test and subjective symptoms on the following day.

**Methods:**

Thirteen healthy Japanese adult males with wild-type aldehyde dehydrogenase (ALDH)2 (*ALDH2* *1/*1) were included in this study. The trial design was a randomized, 2×2 crossover study in which subjects drank (1) *sake* equivalent to 1.3 g/Kg body weight of pure alcohol (Control group) and (2) the same amount of *sake* and 15 mL/Kg body weight of water (Water group) intermittently, each at a constant pace over 1 hour. Before drinking, immediately after drinking, and at various times up to 15 hours after, we measured ethanol and acetaldehyde concentrations in the expired air, administered the Digit Symbol Substitution Test (DSST), and evaluated the subjective intoxication symptom score (VAS). Breath ethanol and acetaldehyde concentrations were determined by sensor gas chromatography. Differences in breath concentrations and pharmacodynamic indices between the two groups were evaluated using a linear mixed-effects model with fixed effects for the test beverages and the number of administrations, and random effects for the subjects.

**Results:**

No differences were observed between the two groups in the kinetics of exhaled ethanol and acetaldehyde. The DSST scores were similar between them. The VAS assessed subjective symptoms of facial flushing, headache, nausea, concentration, sleepiness, and mood elevation, and no differences were found between the two groups.

**Conclusion:**

The intermittent addition of drinking water during ethanol consumption did not significantly alter ethanol disposition or pharmacodynamic parameters, suggesting it did not affect hangover symptoms.

## Introduction

Drinking alcohol has historically and internationally been an ingrained part of society’s culture, contributing to the promotion of human interaction and culture, as well as to stress reduction. On the other hand, inappropriate alcohol consumption has created many problems for people’s health and social life; a WHO report estimates that 2.6 million people worldwide died due to alcohol consumption in 2019, and 116 million DALYs (Disability-Adjusted Life Years) were lost (World Health Organization, 2024).

Often associated with heavy drinking behavior is the so-called hangover. This hangover was defined in 2016 as “a combination of mental and physical symptoms experienced the day after a single bout of heavy drinking, beginning when the blood alcohol concentration (BAC) approaches zero” (Lantman et al., 2016). Subsequent studies have shown that 1) some people experience a hangover even without heavy drinking, 2) the next day is not a limited period, and 3) mental and physical symptoms alone do not describe the hangover. So, it was redefined as “a combination of negative mental and physical symptoms that one would experience after consuming alcohol, beginning when the blood alcohol concentration approaches zero” (Arnoldy et al., 2020). In addition to subjective symptoms such as headache and nausea in the state of hangover, the day after drinking (McKinney, 2010), some reports suggest possible endocrine changes in the form of increased plasma arginine vasopressin, renin activity, aldosterone concentration, and cortisol concentration (Linkola et al., 1978; Linkola et al., 1979), and an association between hangover severity and immune factors (Penning et al., 2010). Furthermore, the possibility of impaired motor vehicle operation and psychomotor function has been pointed out (Verster et al., 2014; Ayre et al., 2022), and there is concern that the effects on people’s social lives may be significant.

Several research reports have been published on methods to alleviate or reduce hangover symptoms. For example, reports have examined the effects of N-acetylcysteine (Coppersmith et al., 2021), supplements containing vitamins B and C (Scholey et al., 2020), and herbal medicines; however, none have shown a significant impact (Wang et al., 2016; Jayawardena et al., 2017). After drinking a high alcohol content drink such as whiskey, one may drink water or a low alcohol content drink as a so-called chaser. It is generally recognized that intermittent hydration during drinking, such as this chaser, may reduce hangover symptoms by compensating for dehydration; however, no randomized crossover studies have examined its effects while controlling for ethanol volume, water consumption, and diet (Mackus et al., 2024). Therefore, we designed and decided to conduct this study.

## Objectives

The purpose of this study was to clarify whether intermittent water intake during drinking causes changes in the breath ethanol and acetaldehyde concentrations, the subjective symptoms, and the psychomotor function, when a healthy adult consumes alcohol to the extent that is expected to present a mild hangover.

## Methods

This study was conducted at the Oita University Hospital Clinical Trial Unit (CTU) from January to March 2023. Before the study commenced, approval was obtained from the Oita University Hospital Interventional Clinical Research Review Committee, and the study was conducted in accordance with the Declaration of Helsinki and the Japanese ethical guidelines for clinical research. The study was conducted after obtaining written consent from the subjects. Before starting the trial, the study protocol was registered in the clinical trial registration system of the University Hospital Medical Information Network Center (UMIN-CTR, No. 000050305; https://center6.umin.ac.jp/cgi-open-bin/ctr/ctr_view.cgi?recptno=R000057281).

### Subjects

Healthy Japanese males were included in the study. Regarding sample size, it was challenging to determine the required number of subjects given the study’s exploratory nature. Referring to a previous study examining physiological changes caused by alcohol consumption in healthy Japanese men (Kido et al., 2016), we set the minimum number of subjects at 6 and the target at 20.

Ethanol is metabolized by alcohol dehydrogenase (ADH) into acetaldehyde, which is further broken down into acetic acid, carbon dioxide, and water by acetaldehyde dehydrogenase (ALDH)2. About 40% of East Asians, including the Japanese, have a genetic mutation (ALDH2*2) that reduces the function of ALDH2 (Yokoyama et al., 2008). In this study, to minimize genotype effects and ensure subject safety, the ALDH2 genotype was restricted to wild-type homozygotes (*1/*1). Other subject selection criteria included an age range of 20 to 50 years, a Body Mass Index (BMI) between 18.0 kg/m^2^ and 30.0 kg/m^2^, and a history of consuming more than 100 g of pure alcohol per day with no significant health issues. Persons with a history of alcohol abuse, a history of alcohol allergy, regular use of medications, liver disease, or any abnormal findings on clinical examination or physician consultation were excluded from the study.

### Trial design

**FIGURE 1:**
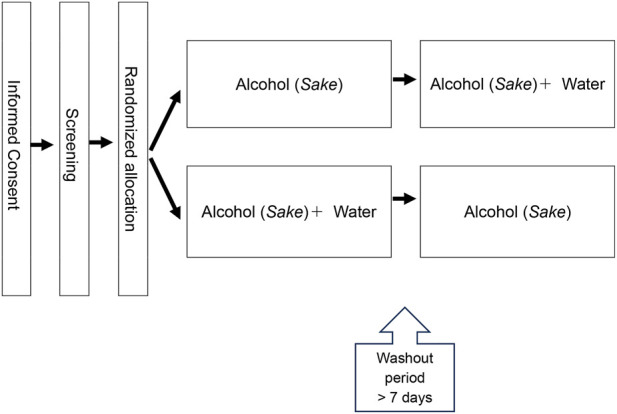
Clinical trial design.

The *ALDH2* genotype of the subject was determined by collecting oral mucosal cells and performing PCR. Subjects participated in two trials, one in which they drank alcohol and the other in which they drank alcohol and water. A crossover design was used in which subjects were randomly assigned to one of the two groups: one group drank only alcohol in the first period and alcohol + water in the second period, and the other group drank alcohol + water in the first period and alcohol only in the second period (Figure 1). At least 1 week was set between the two trials. To control for dietary effects, all subjects were served the same standard dinner at least 2 h before consuming the test beverage. Then, starting at 9:00 p.m., the subjects drank 1.3 g/kg body weight (pure alcohol) of Japanese *sake* (Waka Botan®, Sanwa Shurui Co., Ltd., Oita, Japan) (15.0% alcohol content, v/v) or the same amount of alcohol + 15 mL/kg of mineral water at an even pace over 1 h. Immediately (22:00) and 1 h (23:00) after the end of drinking, breath samples and pharmacodynamic evaluations were collected, and the subjects then slept for approximately 6 h. They woke up 7 h after the end of drinking (5:00). Breath sampling and pharmacodynamic evaluation were conducted thereafter until 15 h after the end of drinking (13:00). The same standard breakfast was served 10 h after the completion of drinking the test beverages (8:00). The consumption of other foods was prohibited during the trial. For drinking water, subjects were given a 550 mL bottle of water after completing the pharmacodynamic tests (22:00) and were allowed to drink freely until the next morning. On the second day of the study, subjects were given a 550 mL bottle of water when breakfast was served and were allowed to drink freely until the end of the study. They were not allowed to drink any other water or beverages.

The Digit Symbol Substitution Test (DSST) was administered to evaluate cognitive function in the following manner: participants were asked to match symbols to as many randomly arranged numbers from 0 to 9 as possible according to a key within 1 min. The number of correct answers was recorded as the score. Subjective symptoms of facial flushing, headache, nausea, concentration, sleepiness, and mood elevation (feeling exhilarated) were evaluated using the Visual Analogue self-rating Scale (VAS), in which subjects themselves marked the corresponding areas on a 100-mm line, and these were repeated at the same time points as the breath samplings.

### Exhaled concentration analysis

The concentrations of ethanol and acetaldehyde in exhaled breath were determined using gas chromatography. After gargling with water, the end-expiratory breath was collected using an exhalation bag (AERoChrome®, Nissha FIS, Inc., Osaka, Japan). The exhaled air (5 cc) was injected into a sensor gas chromatograph unit (SGEA-P3-A, Nissha FIS, Inc.) using a syringe (NORM-Ject®, HENKE SASS WOLF, Tuttlingen, Germany). The detection limits for ethanol and acetaldehyde with this instrument were 0.2 ppm and 5 ppb, respectively.

### Statistical analyses

We employed a per-protocol analysis, focusing on subjects who completed the study protocol. The ethanol and acetaldehyde concentrations in the exhaled breath were analyzed using a linear mixed-effects model with the test beverages (*sake* and *sake* + water), time (first and second periods) as fixed effects, and subjects as variable effects to determine whether there was a difference between the test beverages at each time point.

For the DSST score, the change in score at each time point (i.e., the difference between the DSST score at each time point and the control score before the start of drinking) was used as the objective variable. A linear mixed-effects model with the test beverage (*sake*, *sake* + water) and time (first and second periods) as fixed effects, and subjects as random effects, was used to determine whether the test beverage made a difference. For the VAS score, the score at each time point was used as the outcome variable, and other analyses were conducted as for the DSST score.

All significance levels were set at 5% two-sided, and since this was an exploratory study, multiplicity was not considered. Statistical analysis software was R (Ver. 4.3.1, The R Foundation).

## Results

Consent to participate in the study was obtained from 16 subjects; 2 subsequently withdrew, leaving 14 participants. One participant was excluded from the study because he was unable to drink the prescribed amount of water, and 13 subjects who completed the study were included in the analysis. Background information on the subjects is shown in Table 1. The mean age was 36.2 years, and the mean body mass index (BMI) was 24.0. *Sake* consumption averaged 749 mL (598-967 mL, min-max) and water consumption averaged 1,072 mL (855-1,383 mL, min-max) for each subject.

**TABLE 1:** Demographics of subjects (n = 13).

Age (years)	36.2 ± 9.6
Height (cm)	172.5 ± 6.0
Body weight (kg)	71.5 ± 8.2
BMI (kg/m^2^)	24.0 ± 2.0

Data are shown as Mean ± SD. BMI: body mass index.

**FIGURE 2:**
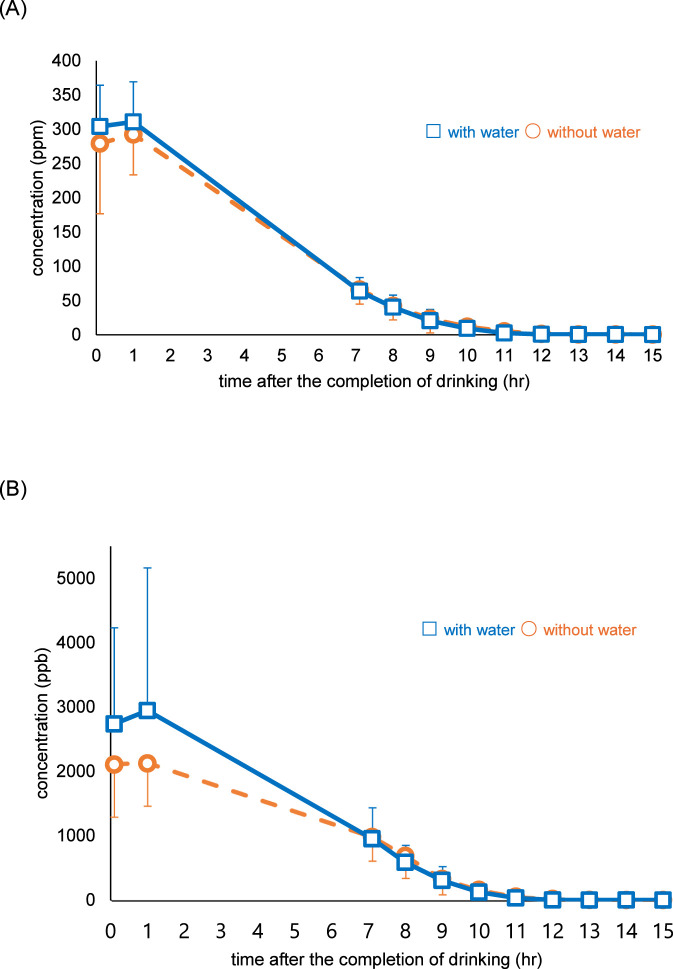
Time-concentration curves for ethanol and acetaldehyde in exhaled air. **(A)** Ethanol. **(B)** Acetaldehyde.

The concentrations of ethanol and acetaldehyde in the exhaled breath were as shown in Figures 2A,B. There was no significant difference between the two groups at any time after the end of drinking (0 h), and the concentrations were similar. However, the point estimates (mean values) of acetaldehyde concentration in the expiratory air immediately after drinking and 1 h later were slightly higher in the water-drinking group, although this difference was not statistically significant.

**FIGURE 3:**
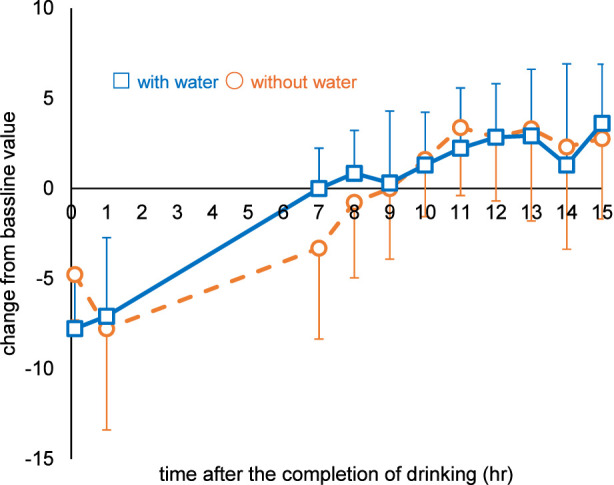
Changes from baseline value in DSST score at each time point.

**FIGURE 4:**
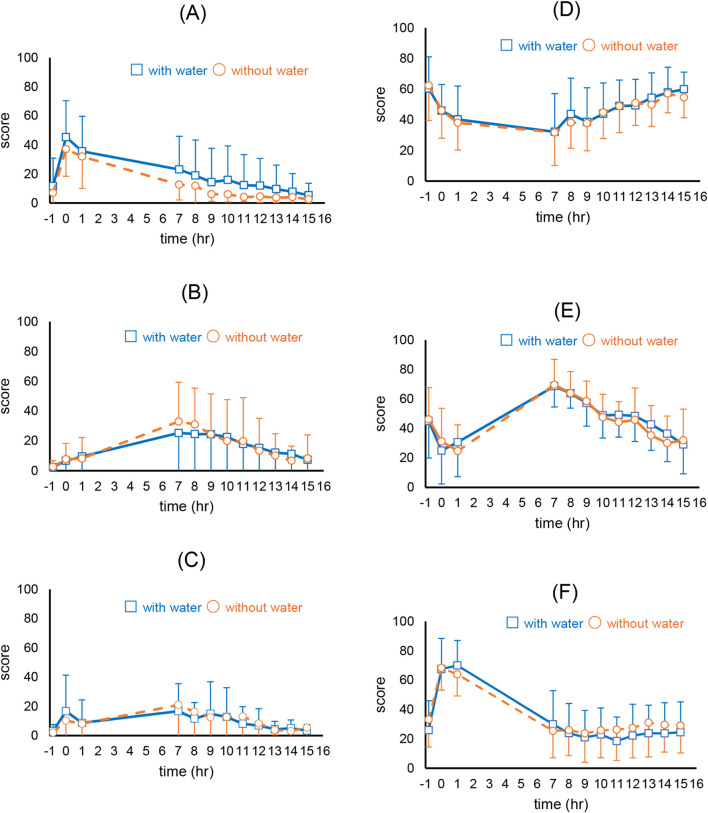
VAS score changes over time curve. **(A)** Facial flushing. **(B)** Headache. **(C)** Nausea. **(D)** Concentration. **(E)** Sleepiness. **(F)** Feeling exhilarated.

The change in DSST score from the control value (before the start of drinking) was lower in the water-drinking group than in the no-water group at 0 h immediately after the end of drinking (p = 0.01) (Figure 3). The DSST scores were higher in the water group at 7 and 8 h after drinking (p = 0.05 and 0.08, respectively), although the differences were not statistically significant; subsequently, they recovered to the pre-drinking level. There were no statistically significant differences between the two groups in any of the VAS assessments (Figure 4).

## Discussions

When the participants drank alcohol, the increase in the scores for facial flushing and elevated mood immediately after and 1 h after the end of drinking indicated that they reached a state of intoxication due to drinking. The increase in the scores for headache and nausea and the decrease in the score for concentration 7 and 8 h after the end of drinking, which was the next morning after sleep, indicated a so-called hangover state. This clinical trial in a 2 × 2 crossover design showed that the exhaled ethanol and acetaldehyde concentrations after drinking to the point of producing a mild hangover were not altered by intermittent water taking, nor did it produce significant changes in psychomotor function or subjective symptoms the next day. The latter is in line with the findings of the previous report (Mackus et al., 2024) on the effects of water drinking. The fact that exhaled alcohol concentrations correlate well with blood concentrations (Jones and Andersson, 2003) suggests that water drinking does not significantly alter blood ethanol and acetaldehyde concentrations. Although the difference was not statistically significant, the DSST score suggested that psychomotor function 7-8 h after the end of drinking (the morning after drinking) may be slightly improved by water drinking.

When humans drink alcohol, ethanol is mainly absorbed in the stomach and upper small intestine, with a bioavailability of approximately 90% (Holford, 1987; Ammon et al., 1996; Zekan et al., 2023). Orally ingested water reaches the small intestine and is primarily absorbed through the lipid bilayers (cell membranes) of the small intestinal mucosal epithelium, facilitated by aquaporins (Zhu et al., 2016). Water addition increases the volume of the interstitial fluid, causing the walls of the stomach and small intestine to stretch. In general, the increased contents in the stomach and intestinal tract stimulate peristalsis and promote excretion (Donnelly et al., 2001). The migration of ethanol and water into the small intestine may have been accelerated, with relatively less absorption in the stomach at the very beginning. Although there was no significant difference, the mean value (point estimate) of acetaldehyde concentrations in expired breath immediately after drinking and 1 hour later was higher when water was added (Figure 2B), suggesting that water intake might have delayed ethanol absorption in the early stages of drinking. Even if there had been a difference, the ethanol and acetaldehyde concentrations in expired breath were almost identical 7 h after the end of drinking, corresponding to the next morning, and the difference was canceled out.

Ethanol is primarily metabolized in the liver, where its hepatic extraction rate is very high, at 0.9 (Holford, 1987; Greenway et al., 1988). Since hepatic clearance is expressed as the product of hepatic blood flow and hepatic extraction rate, substances with high hepatic extraction rates are cleared more rapidly with increased hepatic blood flow. In reality, however, ethanol metabolism is a zero-order reaction because the blood ethanol concentration after drinking exceeds the level at which hepatic alcohol dehydrogenase saturates. Even if the addition of water had increased hepatic blood flow, hepatic ethanol clearance would have remained essentially unchanged. It is possible that adding water may have temporarily increased the body’s water volume and enhanced ethanol’s volume of distribution; however, since the subjects in this study were relatively young and had normal renal function, this effect would likely be negligible. The distribution profile of ethanol and acetaldehyde may differ in older individuals, whose body water content is naturally lower, or in those with renal dysfunction.

Our study has several limitations, including a relatively small sample size. With a sample size of 13 subjects, the power was barely 0.8 when the effect size to be detected at a one-sided significance level of 0.05 was set to 1; therefore, it is possible that smaller effects could not be detected. The participants in this study were all men, so gender differences could not be assessed. A clinical trial involving Han Chinese participants reported that ALDH2 genotype and gender influence ethanol metabolism and psychomotor function, suggesting that gender differences may also exist in the alleviating effects of water on hangover symptoms (Ye et al., 2025).

Another limitation regarding participant characteristics is that racial differences could not be evaluated. For example, while low-activity variants of *ALDH2* are common among East Asians, the genetic frequency of low-activity variants of *ADH1B* is high among Caucasians (Hurley and Edenberg, 2012). Differences in other genetic polymorphisms, as well as in physique and dietary habits, may modify the effects of water consumption. The study’s open-label design may have introduced bias into the pharmacodynamic evaluation. Furthermore, the study design makes it impossible to distinguish between alternating alcohol plus water drinking and drinking double the volume of a low-concentration alcoholic beverage diluted approximately 2-fold. In particular, the water drinking phase involved drinking 15 mL/kg body weight of water in addition to alcohol, resulting in an average fluid intake of approximately 1,800 mL per hour. Several subjects complained of abdominal bloating in the second half of the drinking period, and one participant dropped out because he experienced severe abdominal fullness during the water-drinking test and was unable to drink the prescribed amount. The DSST was significantly lower in the water-drinking group immediately after drinking ended, and the lack of difference in exhaled ethanol suggests that abdominal bloating may have been an unpleasant stimulus that reduced psychomotor function. In addition, the alcohol used in this study was *sake*, a brewed rice liquor that contains sugar and various substances produced during fermentation. Even among Japanese *sake*, yeast and bacterial compositions vary, leading to differences in the metabolic products, such as amino acids and sugars (Akaike et al., 2020). The possibility that the pharmacodynamic evaluations examined in this study were affected by substances other than ethanol, acetaldehyde, and other alcohol metabolites cannot be ruled out. Different results may be obtained with whiskey and *shochu*, which are alcoholic beverages that do not contain sugar. Given these limitations, future studies could evaluate the effects of water consumption on hangover symptoms in greater detail by adopting designs that account for participant diversity, adjusting the amount of water consumed, or conducting comparative studies using distilled spirits as the test beverage.

## Conclusion

Intermittent drinking of water during alcohol consumption hardly changed the concentrations of ethanol and acetaldehyde in the expired breath, suggesting that it did not significantly improve hangover symptoms on the following day. It is generally recognized that drinking water while drinking alcohol reduces hangover symptoms, probably because drinking water naturally limits alcohol intake, resulting in a decrease in the amount and speed of alcohol consumption. However, it was also suggested that drinking water may slightly improve psychomotor function the next morning. This point may warrant further investigation, including, for example, effects on sleep quality and the autonomic nervous system.

## Data Availability

The datasets presented in this study can be found in online repositories. The names of the repository/repositories and accession number(s) can be found in the article/supplementary material.
